# Nutrition knowledge and attitude in medical students of Tabriz University of Medical Sciences in 2017–2018

**DOI:** 10.1186/s13104-019-4788-9

**Published:** 2019-11-21

**Authors:** Neda Dolatkhah, Dawood Aghamohammadi, Azizeh Farshbaf-Khalili, Majid Hajifaraji, Maryam Hashemian, Sepideh Esmaeili

**Affiliations:** 1 Physical Medicine and Rehabilitation Research Center, Aging Research Institute, Tabriz University of Medical Sciences, Tabriz, Iran; 2 Department of Anesthesiology, Faculty of Medicine, Tabriz University of Medical Sciences, Tabriz, Iran; 3 Dept. of Nutrition and Food Policy & Planning Research, National Nutrition & Food Technology Research Institute, Shahid Beheshti University of Medical Sciences, Tehran, Iran; 4 Department of Biology, School of Art and Science, Utica College, New York, USA; 5 Faculty of Medicine, Tabriz University of Medical Sciences, Tabriz, Iran

**Keywords:** Nutrition, Knowledge, Attitude, Medical student

## Abstract

**Objectives:**

In this cross-sectional study among 220 medical students we aimed to determine the nutritional knowledge and attitude of medical students through clinical training courses (externship and internship) of Tabriz University of Medical Sciences, Iran. A nutritional knowledge questionnaire included 51 questions was used to determine the correct, perceived and accuracy of knowledge of the participant in different aspects of nutrition sciences. The nutrition attitude questionnaire included 30 questions. Both questionnaires were confirmed in terms of the validity and reliability for assessing nutritional knowledge and attitude in this sample of Iranian medical students. Nutritional knowledge and attitude were calculated as percentage of correct or appropriate responses.

**Results:**

The correct knowledge was not significantly different among externs and interns (68.20 ± 7.50% and 67.87 ± 6.04% respectively, p = 0.729). Results showed that most of the participants (49.61% of externs and 57.14% of inters) had a poor nutritional knowledge, significantly varied by age (p = 0.035). The attitude index of the subjects was not significantly different among externs and interns (73.36 ± 9.42% and 74.59 ± 9.20%, p = 0.335). Most students (92.7%) had a very appropriate attitude toward nutrition, significantly varied by sex (p = 0.010). These findings indicate that there are multiple deficiencies in nutrition knowledge of medical students.

## Introduction

The emergence of non-communicable diseases (NCDs) is a major challenge to global health [1, 2]. The main causes of the prevalence of chronic NCDs in the world are highly related to lifestyle factors that include unhealthy diet, physical inactivity, cigarette smoking, and excess alcohol consumption [3]. The dangers of diet cause 11.3 million deaths and 241.4 million DALYs according to the perspective of 2013 global disability-adjusted life-years (DALYs) [4]. Dietary behaviors deputize an adjustable and multifaceted lifestyle behavior to modification for the prevention and management of NCDs [5].

People work in the medical profession, that led by physicians often operate as a role model in a community and play a significant role to promote the healthy dietary patterns against non- communicable diseases [6]. Physicians are mostly consulted about health information [7] and compared to dietitians and nutrition counselors are more cost effective [8]. Although patients have accepted physicians as reliable sources of nutrition counseling [9], their expectations are not always met [10, 11]. Several factors, such as lack of time and low patient compliance with diet, contribute to this issue. However, most of physicians has identified lacking training in counseling skills and lacking nutritional knowledge as the most important barriers [12].

Studies indicate that the problem is global [13–15]. Therefore, efforts to promote nutrition education to physicians have never been as urgent as it seems today. So it is proper to measure the nutrition knowledge and attitude in medical students in this altering medical education setting. This will help recognizing any gaps in the medical curriculum and introduce the education policies that are ideal to increase student education outcomes.

This study was designed and conducted to determine the nutritional knowledge and attitude of medical students includes externship (5 years) and internship (7–6 years) training in Tabriz University of Medical Sciences due to the rarity of the studies on nutritional knowledge and attitudes in medical students in Iran, especially in Tabriz University of Medical Sciences.

## Main text

### Method

#### Setting

This cross-sectional study was conducted using observational method and self-administered questionnaires during 2017–2018. A total of 220 medical students were selected from medical school of Tabriz University of Medical Sciences. A trained Nutritionist referred to educational department of medical school then listed the medical students and selected the participants via stratified random sampling method. The criteria for inclusion the study included willingness to participate in the study, being 5–7th year medical student, passing clinical training courses, and passing the basic nutrition course in the basic sciences curriculum. Exclusion criterion was failure to complete the questionnaire. After explaining the objectives of study, obtaining a signed informed consent form, and clarifying the content of the questionnaires, the questionnaires were distributed among the participants.

#### Procedure

Nutritional knowledge questionnaire is a 51-item self-report questionnaire designed by experts of Shahid Beheshti University of Medical Sciences [16]. These questions cover two main areas of basic nutrition [1–12] and clinical nutrition (13–51). Nutritional knowledge was calculated as percentage of correct responses and then ranked [17], which was very good for 85–100% of questions, good for 65–84%, average for 45–64%, and poor for less than 44% of true answers [17]. In this study, three aspects of knowledge including correct knowledge, perceived knowledge and accuracy knowledge were calculated and determined [18]. Correct knowledge level was determined as the sum of the correct responds divided by the number of questions and the report as the correct knowledge percentage [19]. Perceived knowledge was defined as the numbers of “right/wrong” responds to the total “yes/no” questions. In contrast, if the respondent chooses the other options, it seems that he/she has knowledge about this. Therefore, some correct responds to the questions of “Yes/No” were divided by the total numbers of “Yes/No” questions in order to calculate the accuracy of knowledge (what people know) or actual knowledge.

Nutritional attitude questionnaire is a 30-item self-report questionnaire developed by Walsh et al. [20] which was translated and validated according to the published guidelines [21]. The questionnaire included 30 questions: “Nutrition in routine care” [1–8], “Physician–patient relationship” [7–16], “Physician efficacy” [17–22], “opinions about nutrition education in medical school” [23–29], and also an open-ended question [30] for concepts to progress the program. Nutritional attitude was calculated as percentage of appropriate responses.

#### Validity

*Content validity* According the method of Delphi expert enquiry, the questionnaire content validity data were achieved. Ten nutritionists studied the questionnaire in terms of writing and grammar errors and their suitability. The content validity index (CVI) and content validity ratio (CVR) were then calculated [22]. The minimum acceptable value of CVI to confirm each item in both questionnaires was considered at 0.78 [23] and considering the number of nutritionists (n = 10) who reviewed the questions, the minimum acceptable value of CVR was considered at 0.62.

*Face validity* The knowledge questionnaire and translated attitude questionnaire were presented to two separate teams of nutritionists and medical students to determine the face validity.

*Construct validity* Factor structure of the questionnaires was measured by expletory factor analysis (EFA) and using principal component analysis and viramax rotation. Factor loadings of higher than 0.40 were deliberated as descriptive of a meaningful association between item and questionnaires. In this process, the Kaiser–Meyer–Olkin (KMO) index was evaluated and the Bartlett test was performed before determining the EFA.

#### Reliability

The test–retest validity was used to measure the stability of the questionnaire using an intraclass correlation coefficient (ICC) estimate. Thirty students filled the questionnaires twice with 20 days interval. Kuder-Richardson 20 score was considered to assess internal consistency of the questionnaires, and the minimum acceptable value was considered at 0.7 [24].

#### Statistical analysis

The frequency and percentage of categorical variables were determined. Chi square test was used to estimate the association between two categorical data. To determine the correlation between two quantitative variables, Pearson statistical test was used. All analyzes were performed using SPSS software version 17. The significance level of 0.05 was considered as statistically significant.

### Results

The demographic data of the participants are shown in Table 1.

**Table 1:** Socio-demographic characteristics of medical students

Variable	Mean ± SD or n (%)
Age	24.51 ± 1.64
Sex	
Male	96 (43.6%)
Female	124 (56.4%)
Marital status	
Single	170 (77.3%)
Married	47 (21.4%)
Other	3 (1.4%)
The amount of professional study per day	
Less than 60 min	71 (32.3%)
60–120 min	75 (34.1%)
Above 120 min	74 (33.6%)
Interest in nutritional issues	
Very low	21 (9.5%)
Low	26 (11.8%)
Average	114 (51.8%)
High	35 (15.9%)
Very high	24 (10.9%)
Passing a specific nutrition course or class out of the curriculum	
Yes	21 (9.5%)
No	199 (90.5%)
Having a disease that needs a special diet	
Yes	15 (6.4%)
No	205 (93.6%)
Type of disease	
PCOS	3 (20%)
Diabetes	1 (6.7%)
Fatty liver	2 (13.3%)
Obesity	8 (53.3%)
Cancer	1 (6.7%)

#### Validity and reliability the instruments

Following the Delphi round, expert ideas was reasonably uniform. All items in both questionnaires remained because of the criterion of I-CVI > 0.78. The average CVI of the knowledge and Attitude Questionnaire was equal to 0.749 and 0.946, respectively and CVR was equal to 0.801 and 0.917, respectively.

Concerning construct validity, the results of KMO test (0.78 for knowledge questionnaire and 0.92 for attitude questionnaire) more than the recommended index of 0.60 [25] and Bartlett’s test of sphericity (*p* value < 0.05) indicate the adequacy of sample size for performing factor analysis on both knowledge and Attitude questionnaires. All questions in the questionnaires were subject to selection criteria. Viramax rotation was used for factor analysis. The result showed that the knowledge questionnaire had eight factors: factor 1, including items 1, 2, 6, 7, 8, 10, 11 and 12, entitled “Lipid knowledge”, factor 2, including items 13, 19, 20, 21, 46, 47, 48, 49, 50 and 51, related to “nutrition in stress and cancer”, factor 3, including items 3, 14, 30, 31, 32, 33, 34 and 35, related to “nutrition in cardiovascular diseases”, factor 4, including items 15, 16, 17 and 18, related to “weight management”, factor 5, including items 5, 22, 23, 24, 25, 26, 27, 28 and 29, related to “nutrition in gastrointestinal diseases”, factor 6, including items 36, 37, 38 and 39, related to “nutrition in diabetes” and finally factor 7, including items 4, 9, 40, 41, 42, 43, 44 and 45, related to “nutrition renal diseases”. The range of unrotated factor loading in the nutritional knowledge questionnaire was 0.59 to 0.91 (all > 0.45). Accordingly, the attitude questionnaire had four factors. The range of unrotated factor loading in the attitude questionnaire was 0.65 to 0.95 (all > 0.45).

The internal consistency of the knowledge and attitude tool was calculated using Cronbach’s alpha, which for the knowledge and attitude questionnaires were 0.703 and 0.816 (0.765 for question 1–22 and 0.726 for questions 23–29) respectively, with the satisfactory value being 0.70–0.95 [26].

Regarding the reliability evaluation of the knowledge and attitude questionnaire, the correlation coefficient of the knowledge questionnaire was 0.81 in the range of 0.68–0.93. The correlation coefficient of the attitude questionnaire was 0.80 in the range of 0.67–0.94, indicating the reliability of the tool.

#### Nutrition knowledge

The comparison of correct knowledge, perceived knowledge and accuracy of knowledge of participants is shown separately in Table 2. Most students had a weak correct nutrition knowledge (< 68%), with a frequency of 116 (52.3%). Eighty precipitants (36.0%) had average (68–76%), 18 person (8.1%) had good (77–83%) and 6 persons had good (> 83%) correct nutrition knowledge. The relationship between demographic characteristics such as sex and also training course (extern vs. intern) with nutritional knowledge of respondents is shown as Table 3.

**Table 2:** The comparison of different aspects of knowledge and attitude toward nutrition (N = 220)

Variables	Externs (%)	Interns (%)	Total (%)	p-value*
Correct knowledge	68.20 ± 7.50	67.87 ± 6.04	68.07 ± 6.92	0.719
Perceived knowledge	99.95 ± 0.33	99.88 ± 0.895	99.92 ± 0.62	0.373
Accuracy of knowledge	66.26 ± 12.75	62.16 ± 10.77	64.57 ± 12.12	0.014
Attitude				
Total	73.36 ± 9.42	74.59 ± 9.20	73.87 ± 9.33	0.335
Physician and patient relationship	80.32 ± 13.73	82.06 ± 13.48	81.04 ± 13.62	0.353
Nutrition in routine care	72.22 ± 13.17	75.30 ± 13.42	73.50 ± 13.33	0.092
Performance of the physician	67.70 ± 11.17	69.48 ± 12.40	68.43 ± 11.70	0.275
Nutritional education for medical students	83.48 ± 15.25	81.24 ± 11.58	82.56 ± 13.87	0.217

*Obtained from independent samples T test

**Table 3:** Relationship between nutritional knowledge and attitude with demographic characteristics (N = 220)

Characteristics	Knowledge scores				p-value*	Attitude scores			p-value*
	Weak < 68%	Average 68–76%	Good 77–83%	Very good > 83%		Undesirable ≤ 40%	Desirable 41–60%	Very favorable ≥ 61%	
Age									
< 25	55 (46.21%)	49 (41.17%)	9 (7.56%)	6 (5.04%)	0.035	0 (0.00%)	11 (9.24%)	108 (90.75%)	0.233
≥ 25	61 (60.39%)	31 (30.69%)	9 (8.91%)	0 (0.00%)		1 (0.99%)	4 (3.96%)	96 (95.04%)	
Field									
Extern	64 (49.61%)	50 (38.76%)	9 (6.98%)	6 (4.65%)		0 (0.00%)	12 (9.30%)	117 (90.69%)	0.178
Intern	52 (57.14%)	30 (32.98%)	9 (9.89%)	0 (0.00%)	0.261	1 (1.09%)	3 (3.29%)	87 (95.60%)	
Sex									
Male	55 (57.29%)	27 (28.12%)	10 (10.41%)	4 (4.16%)	0.568	0 (0.00%)	12 (12.50%)	84 (87.50%)	0.010
Female	61 (49.19%)	53 (42.74%)	8 (6.45%)	2 (1.61%)		1 (0.80%)	3 (2.41%)	120 (96.77%)	
Marital status									
Single	89 (52.35%)	63 (37.05%)	12 (7.05%)	6 (3.52%)	0.894	1 (0.58%)	10 (5.88%)	159 (93.52%)	0.199
Married	26 (55.31%)	15 (31.91%)	6 (12.76%)	0 (0.00%)		0 (0.00%)	4 (8.51%)	43 (91.48%)	
Other	1 (33.33%)	2 (66.66%)	0 (0.00%)	0 (0.00%)		0 (0.00%)	1 (33.33%)	2 (66.66%)	

*Obtained from Chi Square Test

The most knowledge was in the field of general nutrition with true answer in 86.69 ± 13.44% of participants and the lowest knowledge in the field of fats with true answer in 53.55 ± 13.95% of participants (Additional files 1, 2). Other topics were between these. Also, there was a significant difference between two groups in comparison of nutritional knowledge indices and different domains of nutritional knowledge between externs and interns in dietary fiber (p < 0.001), but in other areas there was no significant difference (p > 0.05).

#### Nutrition attitudes

The comparison of the attitude index in externs and interns is shown separately in Table 2. Comparing students’ attitudes towards nutrition, most students had a very favorable attitude (≥ 60%), with a frequency of 204 (92.7%). Fifteen precipitants (6.8%) had desirable (41–59%) and 1 person (0.5%) had undesirable (≤ 40%) attitude. The relationship between demographic characteristics such as sex and also training course (extern vs. intern) with nutritional attitude of respondents is shown as Table 3.

There was not significant relationship between nutritional knowledge and attitude toward among participants (p = 0.066).

### Discussion

To the best of our knowledge, this was the first study to examine the nutritional knowledge and attitudes of medical students in North West of Iran. We found that the nutritional knowledge was poor in more than half of the participated medical students. The findings were consistent with surveys from other medical universities of Iran and other countries. The results of Abdollahi et al. [16] study in Tehran, Iran show that, nutritional knowledge has been poorly documented for physicians, nurses and nutritionists, especially in the field of clinical nutrition. In another study conducted on medical students of Shahid Beheshti University of Medical Sciences, 73.7% and 75.8% of male and female students had a low and moderate level of knowledge and 98% of students had a low level of nutritional attitude [27].

In this study, the highest knowledge was in the field of basic nutrition with true answer in 86.69 ± 13.44% of participants. Also, the incorrect responds to questions about dietary nutrition knowledge were higher than 80% in both educational courses. Similar the participants of this survey, physicians from Taiwan [28] and Brazil [29] had higher score on basic and general nutrition areas than on more specialized areas.

In this study more than 90% of participants had a very appropriate attitude towards nutrition. The high scores of attitudes among students indicate that they are well prepared to receive relevant knowledge. In line with our findings, in the study conducted by Guan-jin et al. [30] in 2007 on nutritional knowledge and attitudes of medical students, most students had a proper attitude toward nutrition.

### Conclusion

The results of this study indicate that, most of the students of two educational periods have poor nutritional knowledge. The highest area of students’ knowledge was in the field of general nutrition and the lowest in knowledge about fats. The students’ attitude toward nutrition was very appropriate.

## Limitations

The study has some limitations that must be noted. The generalizability of these results should be used carefully i.e. these results represent the perspectives of students from only one university in Iran. Further studies using the longitudinal design based on the students’ academic year are required to determine the change in students’ knowledge and attitudes from the theoretical period to pre-clinical and clinical courses.

## Supplementary information

**Additional file 1.** Scores of 12 areas of nutritional knowledge. Data is presented as percent of participants with true answer for each field of nutritional knowledge.

**Additional file 2.** Questions with the most incorrect answers, separated by educational status. Data is presented as number (percent) of participants with incorrect answer for each question of nutritional knowledge questionnaire.

## Data Availability

All the necessary data are presented herewith. However if needed, raw data on excel format can be availed on reasonable request from the corresponding author.

## Abbreviations

CVI: content validity index
CVR: content validity ratio
EFA: expletory factor analysis
ICC: intraclass correlation coefficient
KMO: Kaiser–Meyer–Olkin
NCDs: non-communicable diseases
