# Supplementary material for: Nutrition knowledge and attitude in medical students of Tabriz University of Medical Sciences in 2017–2018
Source: BMC Res Notes. 2019 Nov 21;12:757. doi: 10.1186/s13104-019-4788-9 (PMC6873575; doi:10.1186/s13104-019-4788-9)
Supplement: Supplementary file 1 — Additional file 1. Scores of 12 areas of nutritional knowledge. Data is presented as percent of participants with true answer for each field of nutritional knowledge. [file 13104_2019_4788_MOESM1_ESM.doc]

| **Nutrition knowledge areas** | **Mean** | **Standard deviation** |
| --- | --- | --- |
| **General nutrition** | 86.69 | 13.44 |
| **Dietary fiber** | 76.82 | 19.58 |
| **Calcium** | 75.91 | 42.86 |
| **Gastrointestinal diseases** | 70.10 | 14.58 |
| **Cardiovascular diseases** | 64.09 | 17.98 |
| **Salt** | 63.55 | 14.81 |
| **Obesity** | 62.39 | 24.07 |
| **Diabetes** | 61.70 | 22.96 |
| **Saturated fats** | 58.90 | 15.07 |
| **Renal Diseases** | 56.52 | 21.91 |
| **Cancer** | 56.21 | 20 |
| **Fats** | 53.55 | 13.95 |
