# Supplementary material for: Nutrition knowledge and attitude in medical students of Tabriz University of Medical Sciences in 2017–2018
Source: BMC Res Notes. 2019 Nov 21;12:757. doi: 10.1186/s13104-019-4788-9 (PMC6873575; doi:10.1186/s13104-019-4788-9)
Supplement: Supplementary file 2 — Additional file 2. Questions with the most incorrect answers, separated by educational status. Data is presented as number (percent) of participants with incorrect answer for each question of nutritional knowledge questionnaire. [file 13104_2019_4788_MOESM2_ESM.doc]

| **Question** | **Externs** | **Question** | **Interns** |
| --- | --- | --- | --- |
|  | n (%) |  | n (%) |
| **Saturated fats are mainly found in which of the following foods?** | 117 (90.70%) | **Saturated fats are mainly found in which of the following foods?** | 84 (92.31%) |
| **What do you think of the type of fat in margarine?** | 111 (86.05%) | **What do you think of the amount of fat in meat?** | 81 (89.01%) |
| **What do you think of the amount of fat in pasta?** | 105 (81.40%) | **What do you think of the type of fat in margarin?** | 77 (84.62%) |
| **What do you think of the amount of fat in meat?** | 104 (80.26%) | **What do you think of the amount of fat in pasta?** | 74 (81.32%) |
| **In patients with swallowing problems, diluted liquids like filtered fruit juice are the best food to swallow** | 91 (54.70%) | **What do you think of the amount of fat in cheese?** | 72 (79.12%) |
